# Antibiotics in the Environment: Occurrence, Enhanced Removal Strategies and Future Prospects

**DOI:** 10.3390/toxics14070637

**Published:** 2026-07-21

**Authors:** Yinglu Tao, Wenjun Xie, Lei Xu, Cailing Shi, Xiangrui Wang, Gaoqi Li, Chufei Yu

**Affiliations:** School of Environmental & Municipal Engineering, Qingdao University of Technology, Qingdao 266520, China; tyl11090713@163.com (Y.T.); xulei24@qut.edu.cn (L.X.);

**Keywords:** antibiotic contamination, removal methods, removal mechanism, environmental media

## Abstract

The widespread occurrence of antibiotics in the environment threatens public health and ecosystem safety. This review summarizes the global occurrence of antibiotic contamination across the different environmental media, i.e., water systems, solid wastes, and soils, and provides a comprehensive analysis of physical, chemical, and biological removal methods, including their mechanisms, application advantages and disadvantages. It is deduced that physical methods aid in antibiotic enrichment, which leads to residual accumulation and fails to achieve complete degradation. In comparison, chemical methods are more efficient and rapid, but they are largely limited by high costs and secondary pollution. Biological methods, despite being appealing due to their low costs and environmental friendliness, may generate and spread antibiotic-resistant bacteria. To overcome the disadvantages of these conventional treatment methods, this review emphasizes the significant potential of integrated antibiotic removal systems, such as coupled advanced oxidation processes (AOPs), physical methods combined with AOPs and chemical methods combined with biological methods, which could achieve superior treatment performance. Future research should focus on optimizing and simplifying coupled systems and developing innovative treatment methods to enhance removal efficiency, reduce operational costs, and minimize secondary toxicity, thereby enabling effective antibiotic pollution remediation. This review summarizes the global state of antibiotic residues and stresses the importance of combined treatment methods for enhancing antibiotic degradation and removal, providing the valuable insights for green and efficient antibiotic treatment.

## 1. Introduction

According to chemical structure, antibiotics are primarily classified into tetracyclines (TCs), sulfonamides (SAs), quinolones (QNs), macrolides (MLs) and beta-lactam. These antibiotics are widely used in the treatment of human diseases and bacterial infections in animals and plants [1]. Due to the overuse or misuse of antibiotics, coupled with the limited metabolism and degradation capacity of living organisms, a large amount of antibiotics is excreted into the environment in urine and feces as parent compounds or active metabolites. Antibiotics are often detected in surface water, underground water, and soil globally, and further gradually accumulate through the food chain, posing an increasing threat to the health of humans, plants and animals [2]. Meanwhile, the presence and persistence of antibiotics has led to the spread of resistance genes (ARGs), which have an adverse effect on the eco-environmental safety and exacerbate ecological risks [3]. Accordingly, the environmental problems caused by antibiotics have become a significant challenge that requires urgent solutions. While antibiotics can be removed by existing approaches, this does not fully deal with the pollution problem. Consequently, developing more effective antibiotic removal technologies remains a pressing need.

While several studies have investigated antibiotic removal methods, they typically focus on a single technology category [4,5,6]. In contrast, this work provides a comparative analysis of physical, chemical, and biological treatment methods, covering removal efficiency, techno-economic feasibility, and practical challenges and limitations of each approach. Furthermore, although combined physical, chemical, and biological methods have shown significant efficacy in antibiotic removal, systematic reviews that categorize and comprehensively evaluate combined methods remain scarce [7]. To address this gap, this work provides a thorough analysis of integrated treatment strategies for antibiotic removal. Additionally, our study reviews the sources, occurrence, and distribution of antibiotics across environmental compartments. By synthesizing the current state of knowledge with a forward-looking perspective on future research directions, this review aims to deepen the understanding of antibiotic residues in the environment and to offer guidance for developing more effective remediation strategies.

## 2. Antibiotic Residues in the Environment

### 2.1. Origins of Antibiotics in the Environment

**Figure 1:**
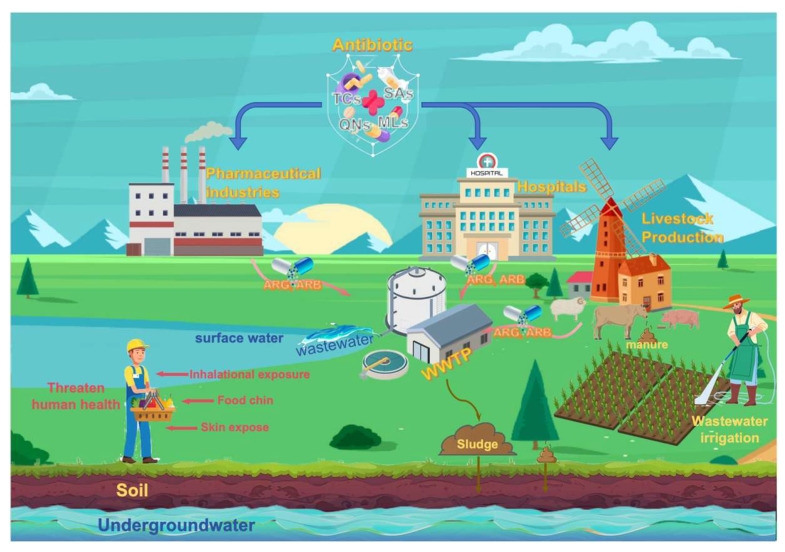
The potential migration routes of antibiotic residues in the environment.

The main sources and environmental fate of antibiotics are shown in Figure 1. The wastewater and wastes discharged from pharmaceutical plants and hospitals are the important sources of antibiotic pollutants, which enter municipal wastewater and are ultimately collected at wastewater treatment plants (WWTPs). Currently, WWTPs have limited effectiveness in removing antibiotic residues; frequent detection of high levels of antibiotic residues often occur in effluent and sewage sludge [8]. When the resulting reclaimed water and sludge are used for irrigation or soil amendment, antibiotics are further dispersed into various environmental media [9]. Furthermore, the application of manure as fertilizer introduces these antibiotics into soil and groundwater. After antibiotics enter the environment, they undergo environmental behaviors such as adsorption, migration and degradation, and are distributed and diffused in the environment for a long period of time and are even enriched through the food chain and the food web, thus generating different degrees of pollution in multiple media.

### 2.2. Current Situation of Antibiotic Residues in the Environment

#### 2.2.1. Water Environment

WWTPs receive untreated hospital and pharmaceutical wastewater, leading to high residual antibiotic levels in effluent, which are ultimately discharged into receiving water bodies. For instance, a WWTP in Kenya showed influent DOX concentrations up to 2900 ng L^−1^, while effluent levels reached 1900 ng L^−1^ [10]. In addition, antibiotics consumed by aquatic animals and livestock are excreted and released into water bodies, either treated or untreated. Consequently, surface water serves as the largest recipient of residual antibiotics. Moreover, antibiotic contamination at varying levels has also been observed globally in groundwater via hydrological circulation. Table 1 and Table 2 summarizes the occurrence concentration of antibiotics in the water environment. In general, SAs, QNs, and MLs are most frequently detected, with higher levels in Asian developing countries than in Europe and North America, likely due to widespread use and inadequate treatment infrastructure [10].

#### 2.2.2. Solid Wastes and Soil

Due to the huge usage of antibiotics, high concentration levels of antibiotics have been found in solid wastes and crop soil across multiple countries (Table 3). Specifically, TCs and FQs are the predominant antibiotics in sewage sludge, owing to their strong adsorption affinity for sludge. Antibiotic concentrations in animal manure are generally higher than those in sludge. TCs and QNs are the most frequently detected, with the highest concentrations of TCs (531~28,317 μg kg^−1^) and QNs (168~16,736 μg kg^−1^) reported in Zhejiang Province, China, far exceeding those in other countries, which is largely attributable to their high consumption [35]. China not only manufactures over 1500 drug varieties and approximately 2 billion kg of active pharmaceutical ingredients annually as the world’s largest producer, but has also witnessed a steady annual increase in domestic antibiotic consumption, with an 82% surge over just five years [36]. Consequently, the antibiotic concentrations reported in China are relatively high. Additionally, irrigation with contaminated surface water, together with the application of antibiotic-laden manure, introduces antibiotics into soil, leading to detectable residues with global concentrations reported as high as 397.6~8400 µg kg^−1^ globally [37].

## 3. Antibiotic Pollution Control and Reduction in the Environment

As the emerging persistent organic pollutants, antibiotics in the environment can inevitably bring great potential harm to the utilization of waste resource, ecological safety, and human health. Hence, antibiotic contamination control has received increasing attention recently.

### 3.1. Physical Methods

#### 3.1.1. Adsorption

**Figure 2:**
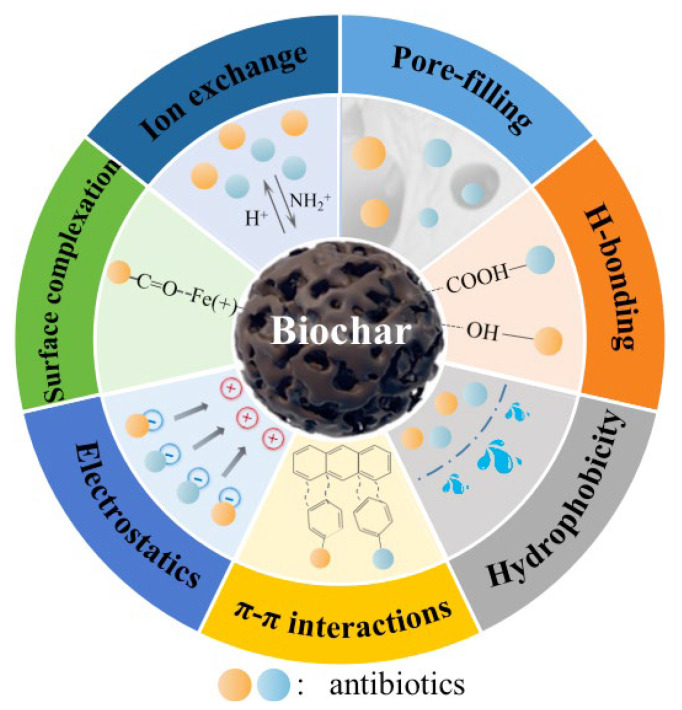
Main adsorption mechanism of biochar on antibiotics.

Adsorption is a viable and attractive method for antibiotic removal, due to its cost-effectiveness, simplicity, and high efficiency. A variety of novel adsorbents have been employed for antibiotics removal, including biochar, activated carbon, clay minerals and other natural/synthetic materials [53]. Due to its low cost, environmental sustainability, and straightforward preparation, biochar is considered a promising adsorbent. The antibiotic removal efficiency of biochar is governed by the physicochemical structure and depends on their specific surface area, pore size distribution, and surface functional groups [54]. As shown in Figure 2, the adsorption of antibiotics by biochar involves multiple interrelated mechanisms. Abundant functional groups on the biochar surface can form hydrogen bonds with antibiotic oxygen atoms, thereby promoting adsorbent–antibiotic binding. The effects of pH and coexisting cations confirm contributions from hydrophobic interactions, Lewis acid–base interactions, and π–π electron-donor–acceptor interactions, with hydrophobic forces between biochar and antibiotic groups likely enhancing the overall affinity. Collectively, these mechanisms, along with pore filling, electrostatic interactions, surface complexation, and ion exchange, constitute the primary adsorption pathways for antibiotics by biochar [55]. To enhance the adsorption performance of adsorbents, some technologies were developed, including acid−base, metal oxide, and metal and non-mental ion modification [56]. For instance, Cheng et al. found that the adsorption capacity of pomelo peel-derived biochar on TC, OTC, and CTC increased from 14.42, 16.77 and 25.85 mg g^−1^ to 476.19, 407.5 and 555.56 mg g^−1^ with KOH modification [57]. Furthermore, activated carbon (AC), especially modified AC, is widely used for antibiotic removal due to its high surface area, adjustable porosity, and strong organic affinity. For instance, Zheng et al. found that modified AC demonstrated superior performance, achieving a 5.07-fold higher NOR adsorption capacity than pristine AC [58].

#### 3.1.2. Membrane Filtration

Based on pore size, membrane filtration can be classified into microfiltration (MF), ultrafiltration (UF), nanofiltration (NF), and reverse osmosis (RO) [59]. Due to molecular size limitations, MF and UF are less effective for antibiotic removal. In contrast, NF and RO demonstrate more pronounced antibiotic removal with more complex separation mechanisms, involving adsorption, size exclusion and charge repulsion [60]. For instance, NF can effectively remove antibiotics such as SMX, CIP, and TC with rejection rates consistently exceeding 80%, while RO achieves even higher removal rates, typically over 95% and even approaching 100% [61,62]. However, fouling remains a significant challenge in membrane filtration. This fouling phenomenon is partly irreversible and categorically negatively affects the efficiency of the membrane separation process. Membrane modification or its integration with other technologies (most often the AOPs) has been pursued to minimize fouling and enhance antibiotic removal efficiency. For instance, Lu et al. modified an NF membrane by incorporating boehmite nanoparticles into the polyamide layer. The results showed that the modified membrane exhibited outstanding removal efficiency for TC (99.2%), excellent antifouling ability and long-term stability [63].

The advantages of removing antibiotics through a physical method are low cost, easy operation, and absence of secondary pollutants. However, the physical method has its own deficiencies, mainly that of no antibiotic degradation and the risk of producing secondary pollution in the subsequent treatment process.

### 3.2. Chemical Methods

#### 3.2.1. Advanced Oxidation

**Figure 3:**
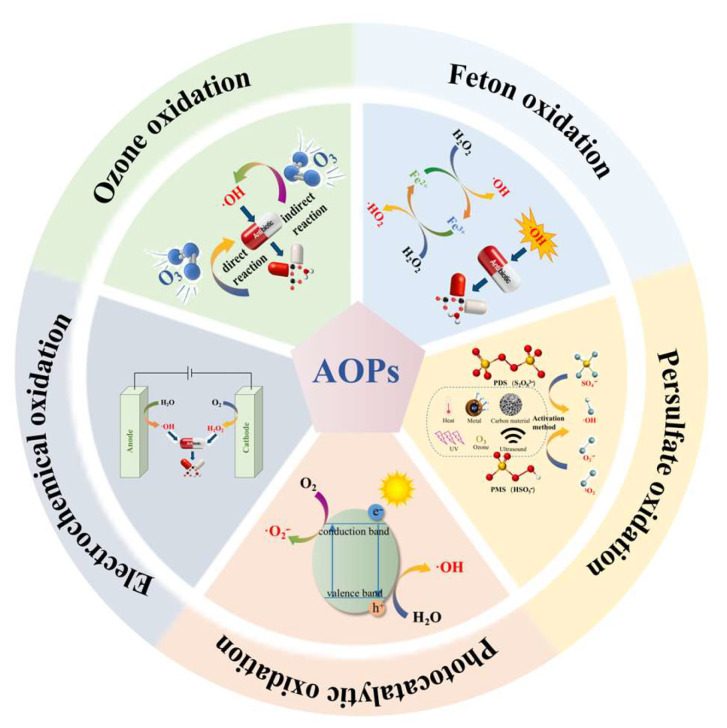
Classification of advanced oxidation processes (AOPs).

Advanced oxidation is a method of transforming or degrading antibiotics through redox reactions. Currently, the commonly used advanced oxidation techniques include ozone oxidation, Fenton oxidation, persulfate oxidation, photocatalytic oxidation and electrochemical oxidation (Figure 3).

1. Ozone Oxidation

Ozone (O_3_) is a strong oxidizing agent with a redox potential of 2.07 V, which can oxidize and decompose organic pollutants in environment. As illustrated in Equations (1) and (2), the ozonation process mainly proceeds via two pathways: the direct oxidation of pollutants (R) by molecular ozone and the indirect oxidation, which primarily involves the generated ∙OH from the decomposition of O_3_, ultimately leading to the breakdown of antibiotics [64]. Anjali et al. found that the degradation of AMX and CIP by ozone could be rapidly degraded and removed within 2 min, and the removal mechanism is mainly through the direct oxidation of ozone molecules [65]. Compared with ozone oxidation alone, the application of catalysts can stimulate ozone molecules to form hydroxyl radicals, especially catalysts containing transition metal oxides (such as MnO_2_, Fe_2_O_3_, Co_3_O_4_, etc.) [66]. For instance, when employed as an ozone catalyst, MnOx/activated carbon composites demonstrated remarkable catalytic efficiency, achieving 80.16% TCH removal within 16 min. The improved performance was attributed to the increased surface area, improved dispersion of manganese oxide, higher density of oxygen vacancies and Lewis acid sites [67]. Similarly, Omid et al. used γ-Al_2_O_3_ as an ozone catalyst and found that the removal rate of CIP by ozone catalytic oxidation was higher than that of ozone treatment [68].

O_3_ + R→ CO_2_ + H_2_O (1)

O_3_ + H_2_O→ 2·OH + O_2_ (2)

2. Fenton Oxidation

The degradation of antibiotics by the Fenton method is mainly based on the rapid reaction of Fe^2+^ with H_2_O_2_ to generate ∙OH with a strong oxidizing ability (E = 2.8 eV), which could attack the active sites of antibiotics and gradually degrade them into small intermediates [69]. When Fe^2+^ reacts with H_2_O_2_ to produce ·OH, Fe^2+^ is oxidized to Fe^3+^, which can form Fe(OH)_3_ coagulation precipitation, thus removing macromolecules of organic matter together (Equations (3) and (4)). In order to overcome the shortcomings of the traditional homogeneous Fenton system, such as the narrow pH range (2.8~3.5) and a large amount of iron sludge production, the heterogeneous Fenton catalysts, such as Fe_2_O_3_, Fe_3_O_4_, CuO, and MnO, have been widely used [70]. An efficient and reusable heterogeneous Fenton catalyst Fe_3_O_4_@S-doped ZnO magnetic composite was synthesized by Wang et al., which can completely degrade OFL in pH of 5.2~9.0 [71]. The catalyst acts as a Lewis acid, creating an acidic microenvironment on the catalyst’s surface, which caused the smooth running of Fenton reaction under alkaline conditions. Similarly, Wang et al. also reported that the Fenton-like catalyst achieved comparable AMX removal efficiency across a wide pH range (3.0, 7.0, and 9.0) [72]. Furthermore, Fenton-like reactions, such as electro-Fenton and photo-Fenton, offer broader applicability, milder reaction conditions, lower cost and higher antibiotic removal efficiency, as detailed in Section 3.2.2.

Fe^2+^ + H_2_O_2_ → ·OH + Fe^3+^ + OH^−^ (3)

Fe^3+^ + H_2_O_2_ → ·OOH + Fe^2+^ + H^+^ (4)

3. Persulfate Oxidation

As illustrated in Equations (5)–(9), persulfate-based oxidants, including Peroxymonosulfate (PMS, ${\mathrm{HSO}}_{5}^{-}$) and peroxydisulfate (PDS, S_2_$O_{8}^{2-}$), can be activated to produce ${\mathrm{SO}}_{4}^{\cdot -}$, ·OH, $O_{2}^{\cdot -}$ and ^1^O_2_. The redox potential of ${\mathrm{SO}}_{4}^{\cdot -}$ is 2.60~3.10 V, exhibiting superior oxidation capacity compared to ·OH. Moreover, ${\mathrm{SO}}_{4}^{\cdot -}$ has a long half-life (30~40 μs), which is 30 times longer than that of ·OH, contributing to prolonged interaction with antibiotics [73]. Persulfate can be activated through various methods, such as heat, carbon material, transition metal ions, ultraviolet light, ultrasound and ozonation [74]. Carbon materials, being low-cost, recyclable, environmentally friendly, and having no extra energy requirement, have been extensively studied for persulfate activation to remove antibiotic pollutants. For instance, Dong et al. found that the persulfate system activated by modified biochar achieved a 91.79% degradation rate for SDZ, outperforming the biochar/H_2_O_2_ system (45.90%) [75]. Additionally, metal catalysts can catalyze the disruption of the S–O bonds to produce ${\mathrm{SO}}_{4}^{\cdot -}$, and many researchers have further combined heat with catalysis to boost pollutant removal. For Instance, Zhou et al. demonstrated that MnO_2_ and heat synergistically activate PDS via chain reactions, producing ${\mathrm{SO}}_{4}^{\cdot -}$ and ·OH for complete SMX degradation. During the processes, heat improved PDS diffusion onto the MnO_2_ surface, promoted the intermediate formation, and facilitated PDS activation by weakening the -O-O- bond [76].

(5) $${\mathrm{HSO}}_{5}^{-}\to {\mathrm{SO}}_{4}^{\cdot -}+\cdot \mathrm{OH}$$

(6) $$S_{2}O_{8}^{2-}\to 2\;{\mathrm{SO}}_{4}^{\cdot -}$$

(7) $${\mathrm{SO}}_{4}^{\cdot -}+{\mathrm{OH}}^{-}\to \cdot \mathrm{OH}+{\mathrm{SO}}_{4}^{2-}$$

(8) O2+e−→O2·−

(9) 2 O2·−+2H+→H2O2+O21

4. Electrochemical Oxidation

Electrochemical oxidation can utilize electricity potential to regulate the redox reaction; H_2_O_2_ and ·OH are produced in a reaction medium when an electric current is applied across two electrodes in water. ·OH is a byproduct of the anodic oxidation of water, while the cathodic reduction of oxygen produces H_2_O_2_ concurrently. Antibiotic degradation (R) primarily occurs at the anode–liquid interface through electron transfer (direct oxidation) or via ·OH generated at the anode (indirect oxidation) [77]. The specific reaction mechanism is shown in Equations (10)–(12). Electrode materials, including widely used metal, carbonaceous, and boron-doped diamond electrodes, substantially influence the antibiotic degradation rate [78]. In addition, different catalysts doped in electrodes also play an important role in the electrocatalytic degradation process of antibiotics. Chen et al. found that Al-doped PbO_2_ electrode exhibited higher electrochemical activity than the undoped one toward chloramphenicol degradation, mainly ascribed to the increase in oxygen evolution potential and radical utilization rate induced by Al^3+^doping [79]. Similarly, Wang et al. found that the La-doped Ti/SnO_2_-Sb/PbO_2_ electrode achieved a higher degradation efficiency of the antibiotic (100% in 10 min) than Ce-doped (89.5%) and undoped (80.8%) electrodes, which was attributed to the creation of more reaction sites by La doping, leading to enhanced oxygen evolution potential and ·OH production capacity [80].

Anode + H_2_O → ·OH + CO_2_ + H^+^ + e^−^ (10)

Anode (·OH) + R → Anode + CO_2_ + H_2_O + H^+^ + e^−^ (11)

O_2_ + 2H^+^ + 2e^−^ → H_2_O_2_ (12)

5. Photocatalytic Oxidation

Photocatalytic oxidation is a type of advanced oxidation process employing light energy and semiconductors as the driving force and catalysts, respectively. Under specific light conditions, the photocatalyst absorbs photons, causing the electrons in the valence band to transition to the conduction band, forming an electron (e^−^)-hole (h^+^) pair. The h^+^ can directly oxidize pollutants or react with H_2_O on the catalyst surface to generate ·OH. Meanwhile, the e^−^ participates in the reduction reaction with oxygen or H_2_O to generate ${\cdot O}_{-2}$ and ·OH (Equations (13)–(15)). In the photocatalytic process, reactive oxygen species such as ·OH, ${\cdot O}_{-2}$, and h^+^ play vital roles in redox reactions [81]. Semiconductor materials such as TiO_2_, ZnS, WO_3_, and SnO_2_ are often used as catalysts during oxidation, and their photocatalytic performance for antibiotic removal can be improved through specific elemental doping. For instance, Cu-doped WO_3_ successfully narrowed the bandgap energy, which enhanced the photocatalytic degradation of TC, achieving 96.8% removal within 60 min [82]. Similarly, Gupta et al. found that the synthesized Ag-doped TiO_2_ nanograins could achieve a photocatalytic CIP degradation efficiency of up to 99.25% within 120 min, surpassing the performance of Au-TiO_2_ (81.48%) and bare TiO_2_ (77.5%) under identical conditions [83]. These are attributed to the coexistence of both Ag^+^ and Ag^0^ species, and the incorporation of Ag^+^ dopants reduced the optical bandgap energy of Ag-TiO_2_, then enhancing light absorption across a broader spectral range.

Photocatalyst + hv →h^+^ + e^−^ (13)

h^+^ + H_2_O → ·OH (14)

e^−^ + O_2_ → ·O_−2_ (15)

The chemical method can degrade all types of antibiotics completely and is rapid and highly efficient in antibiotic removal. However, during the treating process, chemical degradation is prone to cause secondary contamination, and there is the disadvantage of the high cost of chemical reagents. We have compiled a summary of the advantages and disadvantages associated with each advanced oxidation processes, as detailed in Table 4.

#### 3.2.2. Coupled AOP Systems

Electrocatalysis and photocatalysis, which exhibit fundamental affinities with the Fenton process, can be effectively integrated into coupled systems and have shown great potential for enhancing Fenton reaction performance.

1. Electro-Fenton (EF) technology

**Figure 4:**
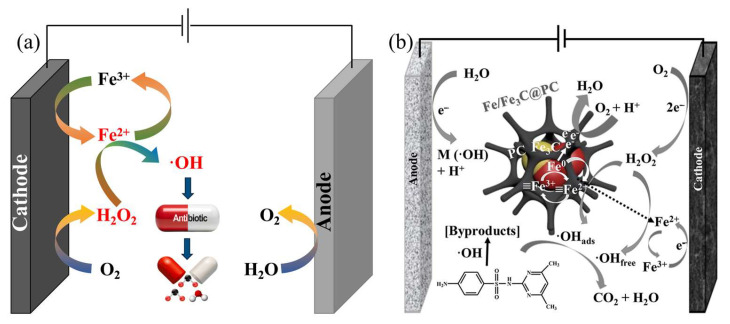
(**a**) Schematic of electro-Fenton reaction for the mineralization of antibiotics. (**b**) The proposed scheme of mechanism of the Hetero-EF process catalyzed by Fe/Fe_3_C@PC catalyst (reproduced with permission from Du et al. [85]).

The EF technology integrates electrochemistry with conventional Fenton reactions, in which H_2_O_2_ is electrochemically produced at the cathode by reducing O_2_ [84]. An iron-containing catalyst facilitates this reaction, producing ·OH for the non-selective oxidation of organic pollutants (Figure 4a). This technology enables controlled in situ H_2_O_2_ generation, eliminating external H_2_O_2_ requirements and reducing operational costs compared to traditional systems. For instance, Du et al. prepared a novel catalyst for the degradation of SMT by heterogeneous EF, which achieved 99% degradation of SMT, within 60 min [85]. The rate constant for SMT degradation in the EF process was 7.17-fold that in the heterogeneous Fenton process. As illustrated in Figure 4b, the degradation of SMT is primarily driven by surface-bound reactions. On the Fe/Fe_3_C@PC catalyst, surface Fe^2+^ sites decompose H_2_O_2_ to form ·OH, which powerfully oxidize SMT to CO_2_ and H_2_O.

2. Photo-Fenton (PF) Technology

**Figure 5:**
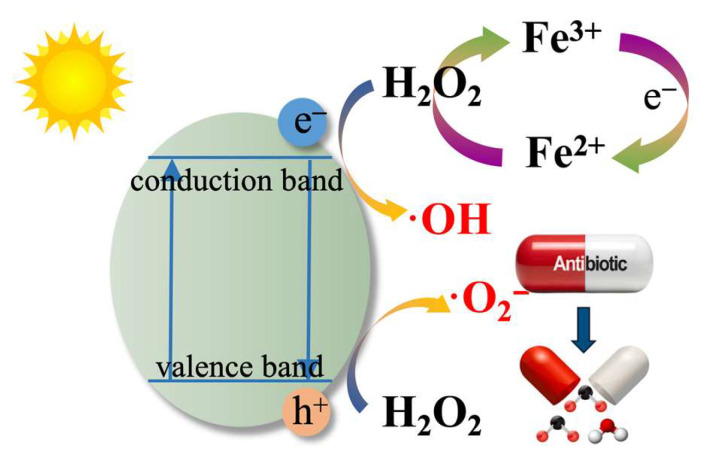
Schematic of photo-Fenton reaction for the mineralization of antibiotics.

The PF process, which leverages light energy to enhance H_2_O_2_ decomposition, improves ·OH yield and accelerates the Fe^3+^/Fe^2+^ redox cycle, boosting the degradation performance of the traditional Fenton reaction while effectively suppressing sludge formation [86] (Figure 5). For instance, Cao et al. developed a heterogeneous photo-Fenton catalyst, LaFeO_3_/BiOI, which achieved a TCH degradation rate of 93.6% within 40 min, compared to 41.2% for single photocatalysis and 83.1% for the Fenton process alone [87]. Results indicated that the existence of the built-in electric field in the LaFeO_3_/BiOI effectively promotes the separation of photogenerated electrons and hole pairs. The photoelectrons activate H_2_O_2_ to generate ·OH and accelerate Fe (III)/Fe (II) conversion. Furthermore, photocatalytic self-Fenton systems, a concept proposed in recent years, offer a distinct advantage over conventional PF systems by generating H_2_O_2_ in situ via photochemical reduction in O_2_ under illumination, thereby enhancing radical generation efficiency synergistically.

3. Photoelectrocatalysis (PEC)

**Figure 6:**
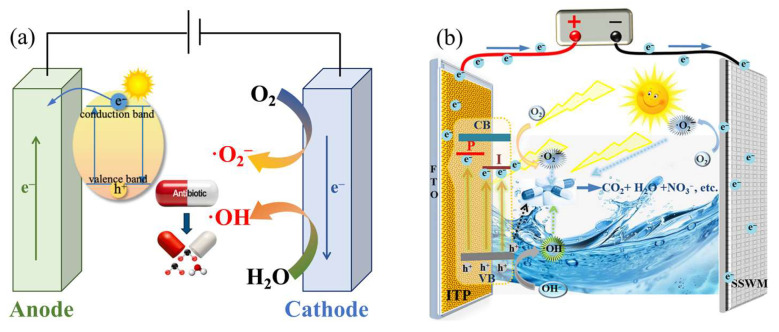
(**a**) Schematic of photoelectrocatalysis reaction for the mineralization of antibiotics. (**b**) Schematic of the enhanced PEC performance of TiO_2_ photoelectrode (reproduced with permission from Liu et al. [90]).

PEC combines the properties of photocatalysis with electrolytic reactions applied in the mineralization of various contaminants. In this technique, a small bias voltage is introduced at the anode to allow the flow of photogenerated electrons to the cathode through an external circuit, which results in the separation of electrons from holes and the generation of more holes and hydroxyl radicals [88] (Figure 6a). Cao et al. reported that the Ag_3_PO_4_/BiVO_4_ photoanode generated a photocurrent over twice that of pure BiVO_4_ under visible light, due to its reduced recombination of photogenerated electron–hole pairs [89]. Similarly, TiO_2_ photoelectrodes prepared by Liu et al. exhibited preeminent PEC performance for the decomposition of TC (99.7%) [90]. As seen in Figure 6b, mechanistic analysis revealed that TC molecules are first adsorbed onto the photoelectrode surface and then rapidly degraded, primarily by hydroxyl radicals and photogenerated holes.

### 3.3. Biological Methods

#### 3.3.1. Phytoremediation

**Figure 7:**
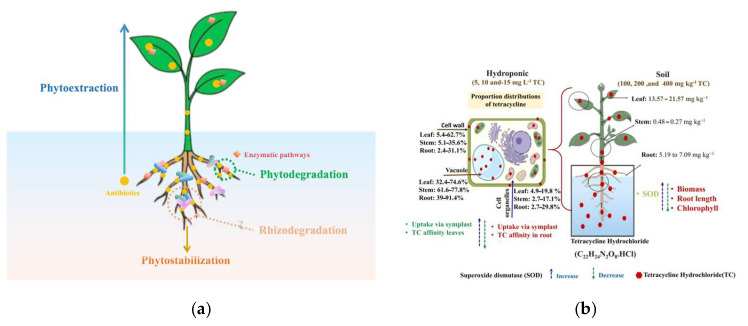
(**a**) Phytoremediation pathways involved in the removal of antibiotic contaminants. (**b**) Mechanisms of TC removal by *Pelargonium graveolens* L (reproduced with permission from Siddiqui et al. [93]).

Phytoremediation, a prominent approach for mitigating antibiotic residues in soil and water, encompasses several key mechanisms: phytoextraction (uptake and accumulation in plant tissues); phytostabilization (immobilization in the rhizosphere); phytodegradation (enzymatic breakdown within plants via oxidation, reduction, or hydrolysis); and rhizodegradation (microbial degradation stimulated by root exudates) (Figure 7a) [91]. Phytoextraction entails the uptake of antibiotic molecules by plant roots, followed by their accumulation in root tissues or translocation to aerial parts. Once internalized, antibiotics may be compartmentalized within vacuoles to mitigate toxicity [92]. For instance, as illustrated in Figure 7b, Siddiqui et al. investigated the potential of *Pelargonium graveolens* L. for removing TC from soil [93]. Their results indicated a high translocation of TC to the aboveground plant parts, with leaf concentrations ranging from 13.57 to 21.57 mg kg^−1^, and predominant localization in the vacuole (32.4~74.6%), exceeding its presence in the cell wall or organelles. Furthermore, the rhizodegradation plays a central role in antibiotic degradation. However, a study by Wei et al. reached different conclusions. While *Hydrilla verticillata* significantly promoted the removal of TC, ENR, and SMX, reducing ARG and MGE abundances by 7.21~86.8%, direct plant uptake contributed only 0.03~6.12%. Instead, the removal relied on microbial regulation mediation [94].

#### 3.3.2. Microbial Degradation

Through screening and domestication, numerous antibiotic-degrading microbes have been identified, most of which can utilize antibiotics as the sole carbon or nitrogen source and employ specific enzyme systems to reduce antibiotic toxicity (Table 5). Microorganisms with the capability of degrading antibiotics mainly belong to the phylum thick-walled bacteria and ascomycetes, but also partly to the phylum actinobacteria, mycobacteria and fungi. However, in practical biological treatment systems, antibiotic degradation is rarely achieved by a single species owing to its susceptibility to environmental fluctuations and lack of competitive advantage, whereas microbial communities, through spatial and functional synergy and division of labor, collectively outperform individual strains [6]. For instance, Yu et al. found that the total oxidizable carbon (TOC) removal rate of a consortium with strain YL1 and YL2 was 38.94%, compared with 29.45% for the single bacterial system. The degradation of SMX was accelerated by the addition of YL2 for its ability to metabolize the key intermediate, 4-aminophenol. Moreover, the mixed bacterial consortium was able to resist SMX at concentrations up to 400 mg L^−1^ and maintained a stable microbial structure under different culture conditions [95]. Similarly, Wu et al. reported that the constructed bacterial consortium achieved a higher TC degradation (81.72%) than the single strain *Raoultella* sp. XY-1 (76.63%); with its application to soil remediation, the relative abundances of most TRGs and MGEs declined [96].

Although removing antibiotics can be done using biotechnologies, the degradation efficiency is easily affected by pH, humidity, nutrient status, and other surrounding circumstances. The mechanism and effectiveness of removing antibiotics in environments should be intensified in future research.

Moreover, the Organization for Economic Co-operation and Development (OECD) and the International Organization for Standardization (ISO) standards are internationally recognized for assessing the biodegradability of organic compounds [97]. However, recent studies have identified several issues in these test systems, which result in false negatives of results, including standardized inoculum pretreatment that may deplete extracellular enzymes and eliminate specialist degraders, the arbitrary 28-day test duration that fails to reach the full extent of possible mineralization of antibiotics, and unrealistic test chemical-to-inoculum ratios [98,99]. Negative results do not prove inherent non-biodegradability, but rather indicate that biodegradation alone is insufficient under standard screening conditions and thus inform the design of combined removal strategies, such as advanced oxidation processes followed by biological degradation, to ensure complete mineralization [7].

**Table 5:** Microorganisms for the biodegradation of antibiotics.

Antibiotics		Strain	Origin	Experimental Condition	Degradation Rate	Reference
Bacterial						
TCs	OTC	*Arthrobacter nicotianae* OTC-16	activated sludge	pH 7.0; 30 °C; 10% inoculum level; 100 mg L^−1^ OTC	98.5% within 8 d	[100]
	CTC	*Pseudmonas* sp. A12	activated sludge	pH 7.0; 30 °C; 5% inoculum level; 2 mg L^−1^ CTC	69.4% within 8 d	[101]
		*Pseudmonas* sp. SF1			81.6% within 8 d	
SAs	SMX	*Pseudomonas* *silesiensis* F6a	wetlands	pH 7.0; 30 °C; 5% inoculum level; 10 mg L^−1^ SMX	76.95% within 144 h	[102]
		*Pseudmonas* sp. A12	activated sludge	pH 7.0; 30 °C; 5% inoculum level; 2 mg L^−1^ SMX	89.6% within 8 d	[101]
		*Pseudmonas* sp. SF1			95.9% within 8 d	
	SMZ	*Bacillus cereus* H38	farmland soil	pH 7.0; 25 °C; 5% inoculum level; 5 mg L^−1^ SMZ	100% within 3 d	[103]
QNs	CIP	*Thermus thermophilus* C419	sludge	pH 6.5; 70 °C; 3% inoculum level; 5 mg L^−1^ CIP	57% within 5 d	[104]
		*Ochrobactrum* sp. YJ17	animal manure	pH 7; 30 °C; 2% inoculum level; 5 mg L^−1^ CIP	63.4% within 14 d	[105]
	OFL	*Thermus thermophilus* C419	sludge	pH 6.5; 70 °C; 3% inoculum level; 5 mg L^−1^ OFL	70% within 72 h	[104]
Fungi						
QNs	CIP	*Trichoderma asperellum*	pure strains	pH 4.9; 25 °C; 200 μg L^−1^ CIP	82% within 13 d	[106]
	OFL			pH 4.9; 25 °C; 200 μg L^−1^ OFL	44% within 13 d	
		*Trichoderma harzianum*			32% within 13 d	

### 3.4. Critical Analysis of Conventional Removal Methods

Physical methods are employed for antibiotic enrichment. Adsorption achieves moderate to high efficiency (50~85%), is readily scalable for decentralized or small-scale systems, and is economically and environmentally favorable, particularly when adsorbents are derived from waste biomass. In comparison, membrane filtration routinely attains higher efficiency (>80~95%), with NF and RO achieving nearly total removal [107,108]. However, owing to its high capital and energy expenditures, membrane filtration is more suitable for high-value wastewater, such as hospital or pharmaceutical effluents. Furthermore, it generates concentrated antibiotic-laden brines that require careful disposal or further treatment.

Chemical methods are generally more efficient and rapid. Ozonation and electrochemical oxidation achieve the highest removal rates (80~90%) within 10~45 min, whereas conventional Fenton and persulfate oxidation are effective but slower (30~60 min) and suffer from narrow pH applicability and excessive iron sludge production [66,109]. However, ozonation and electrochemical oxidation demand the highest energy input, whereas photocatalysis offers a sustainable route with low energy consumption and minimal secondary pollution. Nevertheless, its practical application is hindered by poor stability, limited light penetration, and difficult catalyst recovery.

Despite their appeal owing to low operating costs and environmental friendliness, biological approaches for antibiotic elimination face significant challenges. Microorganisms are sensitive to environmental fluctuations, including pH, temperature, excessive salinity, and heavy metal content; their metabolic activity and antibiotic uptake can be inhibited by such changes [110]. Moreover, the biodegradation process may promote the emergence of ARGs and ARB. Concurrently, sustaining microbial activity in complex waste streams is difficult, which severely restricts scale-up beyond laboratory or pilot scales. Table 6 summarizes advantages and disadvantages of physical, chemical and biological methods.

Furthermore, the economic aspect of these removal methods should not be ignored. AOPs entail high costs, primarily associated with UV irradiation, electrical energy, and chemical reagents. According to previous studies, the cost of commonly used photocatalysts is approximately 1100 $/ton for ZnO, and 1900 $/ton for TiO_2_, with H_2_O_2_ at $500/ton and sodium persulfate at $1400/ton [111]. In addition, electrochemical oxidation requires no chemical reagents, while the consumption of electrical energy and the use of electrode materials constitute major cost factors. Similarly, although ozonation treatment requires fewer reagents, the high energy consumption of ozone generators increases the overall operating cost. In contrast, adsorption has a lower operational cost. Biochar is the best recommended sorbent among others in terms of cost, as the synthesizing cost is only $ 0.2–0.5 per kg [112]. Therefore, to reduce energy consumption and costs, future research should focus on optimizing process parameters to improve oxidant efficiency and developing cost-effective catalysts and electrode materials. Additionally, designing stable and recyclable oxidants and catalysts will minimize replacement frequency and reduce processing costs.

## 4. Combined Methods

Combined methods, which integrate the advantages of physical, chemical, and biological technologies, have demonstrated high efficiency in removing antibiotics from the environment and overcome the limitations of individual treatment processes.

### 4.1. Physical Method-Combined AOPs

#### 4.1.1. Adsorption-Combined AOP Systems

**Figure 8:**
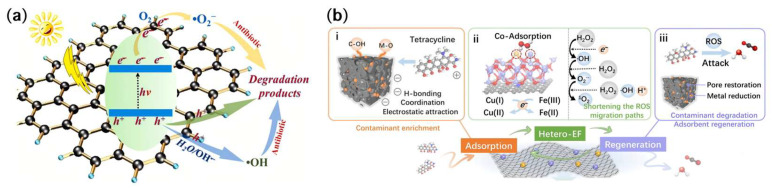
(**a**) Possible photodegradation mechanism of antibiotics over NiCr-LDH/PSB composite (reproduced with permission from Feng et al. [114]). (**b**) Schematic diagram of the Fe-Cu@BPC/A/EF synergy process (reproduced with permission from Ren et al. [115]).

The limitations of adsorption methods such as finite adsorption capacity and the inability to achieve contaminant mineralization have driven interest in coupled adsorption with AOPs. This integration approach enhances AOP efficacy through contaminant preconcentration, catalytic interface provision, or matrix optimization, and enable thorough remediation of antibiotics via synergy [113]. For example, Feng et al. constructed a novel peanut shell biochar anchored NiCr-LDH (NiCr-LDH/PSB) photocatalysts for photodegradation of OFL and TC [114]. Appropriate PSB compositing enhances NiCr-LDH/PSB photodegradation by 3.5-fold for OFX and 2.3-fold for TC relative to NiCr-LDH. As illustrated in Figure 8a, the enhancement of photocatalytic performance arises from PSB’s conjugated π-system and morphology, which broaden light absorption, while PSB acts as both an electron bridge and acceptor to suppress charge recombination and increase active sites. Moreover, as illustrated in Figure 8b, an Fe–Cu@biochar composite synergistically couples adsorption with heterogeneous EF oxidation, achieving >99% TC removal within 20 min with 70.8% lower energy consumption than the non-adsorption system [115]. The adsorbed TC shortens diffusion distances of radicals and non-radical species to boost degradation, while the concurrent degradation regenerates pore structures and adsorption sites, thereby sustaining catalytic performance.

#### 4.1.2. Membrane-Combined AOP Systems

**Figure 9:**
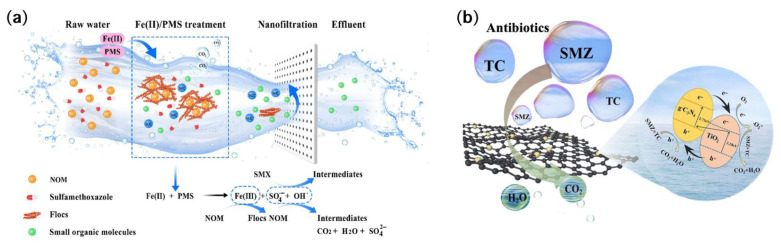
(**a**) The mechanism of Fe(II)/PMS coupled with NF for SMX removal (reproduced with permission from Bai et al. [117]). (**b**) Removal of SMZ and TC using g-C_3_N_4_/TiO_2_ photocatalytic membrane systems (reproduced with permission from Liu et al. [118]).

Membrane filtration suffers from low rejection of small-molecular-weight antibiotics and membrane fouling. Integrating membranes with AOPs addresses these issues through in situ oxidative degradation on/within the membrane, which prevents pollutant accumulation while the synergy between degradation and filtration concurrently mitigates fouling and extends operational life [116]. As illustrated in Figure 9a, Fe (II)/PMS oxidation coupled with NF treatment was extremely effective in degrading SMX, achieving a 92% removal rate, with ${\mathrm{SO}}_{4}^{\cdot -}$ playing a more important role than ·OH. Fe(II)/PMS significantly alleviated membrane fouling and improved the NF membrane flux because the concentration of organic matter decreased [117]. Moreover, photocatalytic membranes integrate photocatalysts and could enhance oxidation and separation efficiency. Liu et al. synthesized membranes containing the g-C_3_N_4_/TiO_2_ photocatalysts, significantly improving the porosity, hydrophilicity and permeability of the membranes. The visible light absorption of g-C_3_N_4_ accelerates electron transfer and promotes the separation of electrons from holes, while the generated h^+^ serves as the primary oxidative species, both mechanisms synergistically contributing to the enhanced removal of SMZ and TC (Figure 9b) [118].

### 4.2. Combined Chemical with Biological Methods

#### 4.2.1. Microbial Fuel Cells

**Figure 10:**
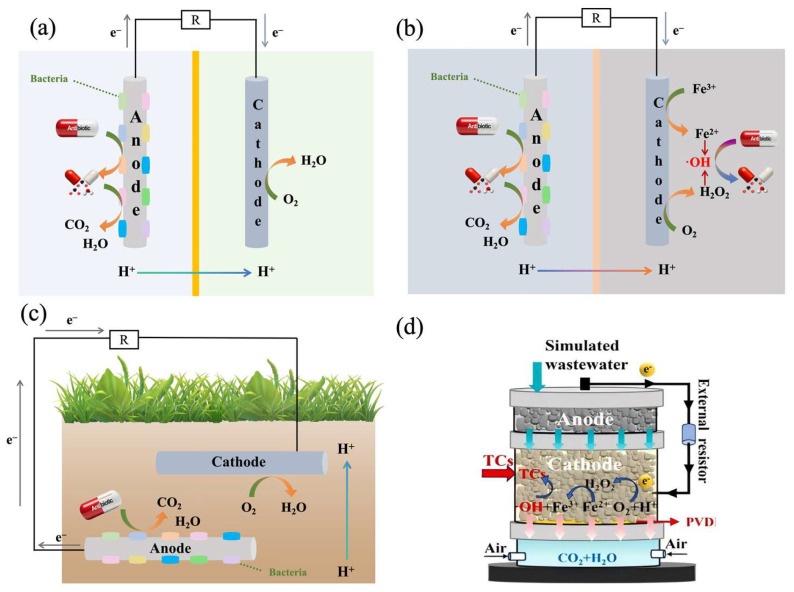
(**a**) Mechanism of microbial fuel cells (MFCs), (**b**) mechanism of MFC coupled with bio-electro-Fenton, (**c**) constructed wetlands and (**d**) membrane bioreactors (reproduced with permission from Li et al. [124]).

Microbial fuel cells (MFCs) are electrochemical devices that utilize the metabolic activity of microorganisms to convert the chemical energy of organic matter into electrical energy, simultaneously realizing degradation of organic pollutants and electricity generation. Through the metabolic activity of anaerobic microorganisms in the anode chamber, MFC can oxidize antibiotics into CO_2_ and H_2_O, while simultaneously releasing electrons [119]. These electrons are transferred to the cathode via an external circuit, while protons move through the proton exchange membrane (PEM) to the cathode chamber (Figure 10a). Xue et al. found that SMX could be thoroughly degraded into less harmful alcohols and methane after the MFC processing, and more than 85.1% of SMX could be degraded within 60 h, demonstrating the strong degradation capacity of MFC [120]. Moreover, MFC can also improve its performance through coupling with other technologies, such as the Fenton system, constructed wetlands (CWs), and membrane bioreactors (MBRs) [121] (Figure 10b–d). For instance, Li et al. constructed a bio-electro-Fenton system to simultaneously degrade TC and LEV wastewater in both anode and cathode chambers. The removal rate of TC in the anode reached 64.54% within 72 h, while that of LEV in the cathode was 91.09% within 24 h [122]. This BEF system can efficiently generate electricity while degrading TC wastewater in the anode chamber, and this bioelectric energy is used to drive the electro-Fenton reaction on the cathode, which degrades LEV wastewater. Additionally, Dai et al. found that the CW-MFC system improved SMX removal from 71.3% to 82.4%, and resulted in a lower abundance of SMX resistance genes, compared to the single CW treatment [123].

#### 4.2.2. Combined Photocatalysis with Biodegradation

**Figure 11:**
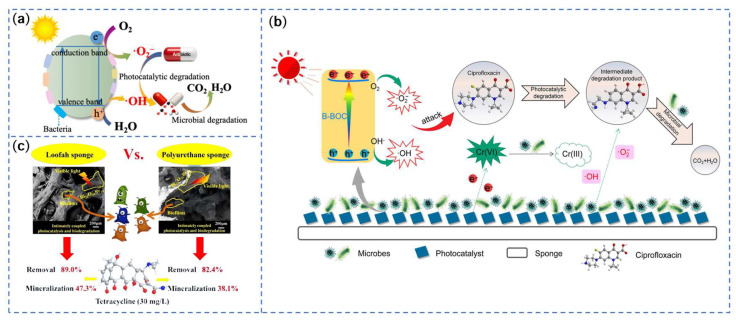
(**a**) Mechanisms of antibiotic degradation by intimately coupled photocatalysis and biodegradation system. (**b**) The synergistic effects of photocatalysis and biodegradation in removal of CIP. (Reproduced with permission from Dong et al. [127]). (**c**) Loofah sponge as a carrier in the ICPB system enhances the biodegradation performance of TC (reproduced with permission from Li et al. [129]).

In recent years, intimately coupled photocatalysis and biodegradation (ICPB) has attracted increased attention in the wastewater treatment. In a ICPB system, the surface of multiple microporous fillers is coated with a catalyst, and then microorganisms attach to the surface of the fillers, resulting in simultaneous photocatalytic and biodegradation synergies in a single reactor [125]. The free radicals (·OH and ${\cdot O}_{-2}$) generated by photocatalysis convert the difficult-to-biodegrade macro-molecular organic matter into easily biodegradable intermediates, which are then degraded by biofilms attached within the filler [126] (Figure 11a). Dong et al. found that the ICPB approach achieved approximately 1.53 times the degradation rate of CIP compared to photocatalysis [127]. Incorporation of B–Bi_3_O_4_Cl (B-BOC) photocatalyst facilitated electron–hole separation, generating ${\cdot O}_{-2}$, ·OH, and h^+^ species that interact with CIP, followed by biodegradation of the photocatalytic products by a protective biofilm (Figure 11b). Similarly, in another study, the removal rate of TCH in water by the ICPB system was 96.0% after 10 h, which was significantly higher than that by the photocatalysis (76.3%) or biodegradation (32.5%) [128]. In addition, the selection of photocatalysts and carriers are the key factors influencing the degradation of antibiotics by ICPB. In comparison to polyurethane, the carrier of a natural three-dimensional structure of loofah sponge in ICPB increased TCH removal rate by 6.6%, and increased the mineralization of the photocatalysis intermediate by 9.2% [129]. As illustrated in Figure 11c, the biocarrier provided a higher loading density and a higher microbial activity of bacteria than that of the polyurethane carrier. In addition, it can regulate the bacterial community to have more favorable members to biodegrade the photocatalysis intermediates.

## 5. Conclusions and Future Research Prospects

This paper elucidates the sources of antibiotic contaminants in the environment. At the same time, the current status and contamination levels of antibiotics are analyzed. TCs, SAs, QNs, and MLs are the most common antibiotics with high detection rates in different environmental media. Additionally, this paper reviews the research and application of physical, chemical, biological and coupling methods in antibiotic pollution control, including the degradation efficiency, removal mechanism, influencing factors, advantages, disadvantages and research prospects of treatment methods. Nevertheless, at present, the problem of antibiotic residues in environment remains unsolved. The following aspects should be considered in future studies:

1. The lack of regulations governing the production, use, and disposal of antibiotics has contributed to rising environmental contamination. Therefore, it is essential to establish monitoring systems and strengthen relevant legislation. Emission standards should be formulated and enforced for key sources such as livestock farming and pharmaceutical wastewater to mitigate antibiotic accumulation in the environment.
2. Physical treatments (e.g., adsorption, membrane separation) removes antibiotics from the environment via enrichment or interception. These methods are simple and low-cost but only concentrate the antibiotics, requiring further treatment for complete removal. The combination of physical methods with AOPs not only achieves the simultaneous enrichment and removal of antibiotics but also creates a synergistic effect that leverages the strengths of both approaches. Future research should focus on coupling physical methods with AOPs or other processes as a pretreatment technology or as a complementary strategy to achieve simultaneous enrichment and removal.
3. Despite the significant advantages of AOPs in antibiotic degradation, challenges including high cost, substantial energy consumption, secondary pollution risks, and operational sensitivity remain. Therefore, developing coupled treatment technologies that combine AOPs with physical or biological methods is crucial to improve removal efficiency and minimize energy consumption. Additionally, AOPs may pose certain negative impacts on the safe utilization of solid waste and soil remediation. Excessive residual oxidants can lead to the loss of organic matter in solid waste, disrupt soil physicochemical properties and alter microbial community structure. Therefore, future AOP applications should optimize oxidant dosage, activation methods, and reaction conditions to balance efficient antibiotic removal with the safe utilization of solid waste and soil ecological security.
4. The biological method is environmentally friendly and cost-effective; nevertheless, it takes a long time to degrade antibiotics and treatment efficiency is highly dependent on environmental conditions. In addition, ARGs and antibiotic-resistant bacteria (ARB) can emerge during the treatment process, which has potential risks to biological safety to a certain extent. Therefore, future research should develop innovative approaches to enhance antibiotic biodegradation, such as construction of microbial consortia and genetically engineered microorganism. Furthermore, it is crucial to strengthen the monitoring of ARGs and ARB during treatment and to adjust the treatment strategy in time, which can reduce the spread of drug resistance.
5. The application of physical, chemical and biological methods has different advantages and disadvantages, while combining methods could compensate for the shortcomings of a single technology. Integrating physical methods with AOPs offers a promising strategy to enhance removal efficiency and reduce costs by leveraging mechanisms such as contaminant preconcentration and matrix optimization. Future work should focus on designing tailored, stable materials and optimizing reactor configurations through modeling to maximize synergy and operational stability. Furthermore, AOPs coupled with biodegradation have also shown considerable promise, as their synergy between catalytic oxidation and microbial metabolism enables efficient antibiotic degradation and enhanced mineralization. Future research should focus on optimizing AOP reactor design, to prevent excessive oxidation of organic matter and enhance subsequent biological degradability, thereby achieving an effective AOP–biodegradation balance. Additionally, novel oxidants should be introduced to overcome the limitations of traditional processes through enhanced selectivity and biodegradability.

## Figures and Tables

**Table 1:** Concentrations of antibiotics in wastewater treatment plants.

Species		Country	Sampling Site	Influent Concentration (ng L^−1^)	Influent Concentration (ng L^−1^)	Reference
TCs	TC	Qatar	Doha	199~319	197~260	[11]
	CTC	Italy	Rome	4487.18	898.72	[12]
	DOX	Kenya	Machakos	2500~2900	1100~1900	[10]
SAs	SMZ	USA	Gwinnett	1200~3400	35~140	[13]
		Finland	Jyväskylä	202	130	[14]
	SDZ	Greek	Volos	846	194	[15]
QNs	OFL	China	Wuhu	49.0~1124.8	231.5~683.5	[16]
	CIP	USA	Gwinnett	430~1100	1~10	[13]
		Qatar	Doha	234~2543	238~1723	[11]
		Kenya	Machakos	1200~2000	100~700	[10]
MLs	CAM	China	South China	26~1854	4.79~637.1	[17]
		Finland	Helsinki	50~327	140~219	[18]

**Table 2:** Concentrations of antibiotics in surface water and groundwater.

Species		Country	Sampling Site	Concentration (ng L^−1^)	Reference
surface water					
TCs	TCs	China	Yellow River	8.25~131.59	[19]
	TC	South Africa	Msunduzi River	158.42~1290.43	[20]
	DOX	Kenya	Mitheu river	600~800	[10]
SAs	SAs	China	East China	3.63~203.65	[21]
	SMZ	South Africa	Umgeni Rive	360~1100	[22]
		USA	North Carolina	5.97~14.54	[23]
		German	Thulsfelde	147	[24]
	SMX	German	Thulsfelde	114	
	SDZ	China	Hai River	54.6~505	[19]
		UK	Thames river	5.0~5.4	[25]
QNs	NOR	Kenya	Mitheu river	590~610	[10]
	CIP	China	North China	62.04~641.3	[26]
		Kenya	Mitheu river	1200~1400	[10]
		Brazil	Três Marias reservoir	3.3~17.7	[27]
MLs	CAM	UK	Thames river	5.7~500	[25]
		Brazil	Curitiba	80~650	[28]
	CLA	South Korea	Han River	79~223	[29]
Groundwater					
TCs	TC	China	North and South China	10.3~207.1	[30]
	TC	Spain	Osona catchment	40~140	[31]
	OTC	China	North and South China	13.1~517.6	[30]
	CTC	Spain	Osona catchment	64~365	[31]
	DOX	Spain	Osona catchment	21~2400	
SAs	SMZ	Germany	Baden-Württemberg	410	[32]
QNs	OFL	China	Wuhan	4.0-215.4	[33]
	NOR	China	North and South China	25.2~142.0	[30]
	CIP	Spain	Barcelona	443	[34]
MAs	ETM	China	North and South China	13.0~377.8	[30]
		Romania	Cluj-Napoca	258.3	[34]

**Table 3:** Concentrations of the antibiotics in solid wastes and soil.

Species		Country	Sampling Site	Concentration (μg kg^−1^)	Reference
Sewage sludge					
TCs	TC	Nigeria	Lagos	179.58~310.2	[38]
	OTC	Nigeria	Ibadan	364.81	
		Brazil	Porto Alegre	62~290	[39]
SAs	SAs	China	Shandong	0.14~29.6	[40]
QNs	QNs	China	Beijing	989~10,096	
	OFL	Canada	Ontario	150~3200	[41]
	CIP	Canada	Ontario	1780~16,000	
		Nigeria	Lagos	112.03~674.0	[38]
		Swedish	Göteborg	1600~11,000	[42]
Animal manure					
TCs	TCs	China	Beijing	531~28,317	[43]
	TC	USA	New York	30~420	[44]
	OTC	China	Zhejiang	3160~5510	[35]
		Spain	Baix Empordà	20~6700	[45]
	CTC	USA	New York	7~107	[44]
	DOX	Netherlands	Wageningen	324~4500	[46]
		Belgium	Flanders	17.9~13,632.1	[47]
SAs	SDZ	China	Zhejiang	3430~7620	[35]
		Netherlands	Wageningen	80~216	[46]
QNs	QNs	China	Beijing	168~16,736	[43]
	CIP	Spain	Baix Empordà	54~2900	[45]
MLs	TYL	Netherlands	Wageningen	10~516	[46]
		Belgium	Flanders	17.3~5599.0	[47]
Soil					
TCs	TCs	China	Beijing	53~430	[43]
	OTC	China	Tongshan	397.6~8400	[37]
		Pakistan	Kohat	7.44~29.22	[48]
	CTC	China	Zhejiang	1148.5	[49]
		China	Shenyang	8.29~1590.16	[50]
	DOX	Malaysia	Sendayan	193~537	[51]
SAs	SDZ	China	Shenyang	1.93~760.09	[50]
QNs	QNs	China	Beijing	51~649	[43]
	CIP	Switzerland	Zurich	270~400	[52]
	NOR	Switzerland	Zurich	270~320	[52]
MLs	TYL	Malaysia	Linggi	187~1171	[51]

**Table 4:** The advantages and disadvantages of different advanced oxidation processes.

AOPs	Main Reactive Species	Advantages	Disadvantage
Ozone oxidation	O_3_, ·OH	High oxidation rate Simple operation Low secondary pollution	High energy consumption High operational costs Strong selectivity
Fenton oxidation	·OH	High oxidation rate Simple operation Low selectivity	Limited to the acidic condition Excessive iron sludge production Low H_2_O_2_ utilization rate
Persulfate oxidation	${\mathrm{SO}}_{4}^{\cdot -}$, ·OH, $O_{2}^{\cdot -}$, ^1^O_2_	Stronger oxidation capacity Wide pH range	Activation is energy-intensive and high costs Poor room-temperature activation Poor catalyst stability
Electrochemical oxidation	·OH	Low secondary pollution Strong applicability Require fewer reagents	High energy consumption Reactor design is complex Electrodes are corrosion-prone
Photocatalytic oxidation	·OH, h^+^, ${\cdot O}_{-2}$	Low secondary pollution Simple operation	Low utilization of light energy Difficulty in catalyst recovery

**Table 6:** The advantages and disadvantages of different treatment methods.

Method	Treatment Efficiency	Advantages	Disadvantage	Reference
Physical	50~99%	Low cost Low energy consumption No by-products	Transfer rather than degradation High regeneration costs Secondary waste disposal burden	[107,108]
Chemical	80~99%	Rapid and efficient degradation Wide application range Simple and mature operation	Potential formation of toxic transformation products or by-products High energy and chemical reagent consumption	[4,109]
Biological	32%~100%	Low cost Simple operation Environmentally friendly	May promote the emergence of ARGs and ARB Sensitive to environmental conditions Long degradation period	[103,106]

## Data Availability

No new data were created or analyzed in this study. Data sharing is not applicable to this article.

## Abbreviations

The following abbreviations are used in this manuscript:

AMX	Amoxicillin
AZM	Azithromycin
CAM	Carrimycin
CIP	Ciprofloxacin
CLA	Clarithromycin
CTC	Chlortetracycline
DOX	Doxorubicin
ETM	Erythromycin
LEV	Levofloxacin
ENR	Enrofloxacin
NOR	Norfloxacin
OFL	Ofloxacin
OTC	Oxytetracycline
STZ	Streptozotocin
SDZ	Sulfadiazine
SMT	sulfamethazine
SMX	Sulfamethoxazole
SMZ	Sulfamerazine
TC	Tetracycline
TCH	Tetracycline hydrochloride
TYL	Tylosin
